# Self-Assembled Aminated and TEMPO Cellulose Nanofibers (Am/TEMPO-CNF) Aerogel for Adsorptive Removal of Oxytetracycline and Chloramphenicol Antibiotics from Water

**DOI:** 10.3390/gels10010077

**Published:** 2024-01-20

**Authors:** Rabia Amen, Islam Elsayed, Gregory T. Schueneman, El Barbary Hassan

**Affiliations:** 1 Department of Sustainable Bioproducts, Mississippi State University, P.O. Box 9820, Mississippi State, MS 39762, USA; ra1070@msstate.edu (R.A.); iae10@msstate.edu (I.E.); 2 Department of Chemistry, Faculty of Science, Damietta University, New Damietta 34517, Egypt; 3 Forest Products Laboratory, USDA Forest Service, Madison, WI 53726, USA; gregory.t.schueneman@usda.gov

**Keywords:** aminated-CNF, TEMPO-CNF, aerogel, antibiotics, adsorption

## Abstract

Antibiotics are used for the well-being of human beings and other animals. Detectable levels of antibiotics can be found in pharmaceutical, municipal, and animal effluents. Therefore, the treatment of antibiotic contaminated water is of great concern. In this study, we fabricated a sustainable aminated/TEMPO cellulose nanofiber (Am/TEMPO-CNF) aerogel to remove oxytetracycline (OTC) and chloramphenicol (CAP) from synthetic wastewater. The prepared aerogel was characterized using different analytical techniques such as elemental analysis, FTIR, TGA, SEM-EDS, and N_2_ adsorption–desorption isotherms. The characterization techniques confirmed the presence and interaction of quaternary amine -[NR_3_]^+^ and -COOH groups on Am/TEMPO-CNF with OTC and CAP, which validates the successful modification of Am/TEMPO-CNF. The adsorption process of the pollutants was examined as a function of solution pH, concentrations, reaction time, and temperatures. The maximum adsorption capacity was 153.13 and 150.15 mg/g for OTC and CAP, respectively. The pseudo-second order (PSO-2) was well fitted to both OTC and CAP, confirming the removal is via chemisorption. Hydrogen bonding and electrostatic attraction have been postulated as key factors in facilitating OTC and CAP adsorption according to spectroscopic studies. Energetically, the adsorption was spontaneous and endothermic for both pollutants. In conclusion, the efficient removal rate and excellent reusability of Am/TEMPO-CNF indicate the strong potential of the adsorbent for antibiotics’ removal.

## 1. Introduction

Antibiotics’ potent bactericidal and bacteriostatic actions have made them indispensable for the treatment of a broad variety of infectious illnesses in humans as well as animals. Antibiotics can improve feed effectiveness, which in turn raises the worth of live-stock products [1,2]. Antibiotics are notoriously difficult to break down since they include many biologically harmful chemicals. Many antibiotics end up in the environment through urine and feces as a consequence of inadequate absorption and metabolism, where they may cause damage to aquatic life [3]. Wastewater from pharmaceutical factories has also been reported to pollute nearby surface and groundwater with biologically active drugs [4]. Antibiotics have toxic effects on aquatic organisms and can cause chronic toxicity, endocrine disruption, and changes in the genetic composition of microbial communities in aquatic environments [5]. Furthermore, it has been shown that antibiotics in water bodies promote the development and spread of antibiotic resistant genes [6,7].

According to reports, the effluents originating from pharmaceutical manufacturing facilities, intense aquaculture ponds specifically used for shrimp farming, and animal farms specifically raising pigs, have been found to have notably elevated levels of antibiotics, particularly oxytetracycline (OTC), with concentrations reaching as high as 32.0 mg/L [1,8]. Oxytetracycline is also poorly absorbed by animals, therefore, roughly 70% to 90% of it gets released into the water, along with its metabolites. The substantial occurrence of OTC drugs and their subsequent degradation precursors in aquatic ecosystems has led to a notable accumulation, resulting in enduring ecological damage to the water system [9].

Chloramphenicol (CAP) is a commonly utilized antibiotic that belongs to the chlorinated nitroaromatic class. It possesses notable antibacterial characteristics and is regularly observed in various environmental settings [10]. The widespread utilization of this substance in healthcare, veterinary care, and aquaculture activities leads to the accumulation of considerable residues of CAP in water systems. In certain regions, the concentration of CAP may reach up to 10^−6^ g/L [11]. Chloramphenicol possesses the capacity to induce notable deleterious effects on living things, such as the onset of aplastic anemia and gene-toxic malignancy [10,12]. Conventional water treatment methods have limited efficacy for removing OTC and CAP, which is mostly attributed to their high chemical stability, high hydrophilicity, and their resistance to biodegradation under both aerobic and anaerobic conditions [5,13]. Therefore, it is essential to develop an efficient treatment technique for the removal of OTC and CAP from wastewater. Hence, the development of an effective treatment methodology for the elimination of OTC and CAP from wastewater is of utmost importance.

Different techniques are being used for the treatment of antibiotic contaminated water including ion exchange, coagulation, oxidation, chemical precipitation, photocatalytic degradation, etc. [14,15]. However, the use of these strategies is constrained by various disadvantages including high operational cost, formation of secondary pollutants, and chemical consumption. Comparatively speaking, adsorption is considered an efficient, cost effective, accessible, and sustainable approach for water treatment. Different adsorbents have been used for the antibiotics’ treatment including carbon nanotubes [16], metal organic frameworks [17], biochar [18], and activated carbon [19].

Aerogels have been widely recognized as very promising materials for use as adsorbents in the field of water purification. Aerogels are chemically manufactured porous lightweight materials in which the hydrogel is replaced with a gas via freeze drying techniques without causing significant impact on its structure [20]. In comparison to organic aerogels, inorganic aerogels exhibit notable drawbacks, including diminished durability and instability. The use of cellulose nanofibers (CNF) based aerogel has emerged as an environmentally friendly material due to its notable attributes of being derived from renewable sources and environmentally biodegradable [14].

The primary objective of this work is to produce an aerogel material by electrostatic attraction between a quaternary amine group of aminated cellulose nanofibers (Am-CNF) and a carboxyl group of TEMPO cellulose nanofibers (TEMPO-CNF). The TEMPO oxidation process is used to selectively form carboxyl (COOH) groups by oxidizing the alcoholic hydroxyl groups on carbon no. six of glucose units in the CNF. The above-mentioned carboxyl group may be used for the purpose of electrostatic bond creation with quaternary amine groups, or alternatively, it can aid in the facilitation of the development of ionic connections [21,22]. It is anticipated that the formulated aerogel will demonstrate efficacy against both oxytetracycline and chloramphenicol antibiotics. To enhance the mechanical properties and broaden the potential uses of quaternary amine-based aerogels in the context of wastewater treatment, a unique method for producing quaternary amine-based/TEMPO cellulose nanofibers (Am/TEMPO-CNF) aerogels has been introduced for the first time. The effective fabrication of the Am/TEMPO-CNF composite is achieved by employing strong electrostatic attraction between the carboxyl group of the TEMPO-CNF and the quaternary ammonium cation of Am-CNF, as well as the hydrogen bonding of hydroxyl groups.

The OTC and CAP are chosen as representative antibiotics to evaluate the influence of Am/TEMPO-CNF on antibiotics’ sorption. A series of batch experiments have been conducted to examine the adsorption behavior of Am/TEMPO-CNF involving the evaluation of the following parameters: effect of contact time, initial antibiotic concentration, adsorption thermodynamics, regeneration potential, and adsorption mechanisms. The finalized data was fitted into kinetic and isotherm models to study the antibiotic adsorption behavior using Am/TEMPO-CNF.

## 2. Results and Discussion

### 2.1. Characterization of Am/TEMPO-CNF

The elemental analysis of Am/TEMPO-CNF was undertaken to evaluate the successful modification of CNF. The difference between the nitrogen content in the CNF, Am-CNF, and Am/TEMPO-CNF can be observed clearly in Table 1. Both Am/TEMPO-CNF and Am-CNF have higher nitrogen content (1.26% and 2.93%, respectively), compared to CNF, which confirms the successful amination step for the CNF.

**Figure 1:**
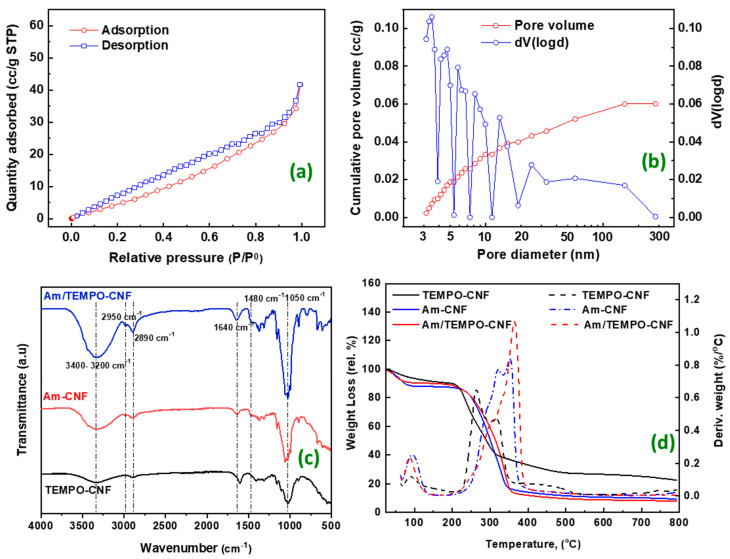
(**a**) Am/TEMPO-CNF nitrogen adsorption–desorption isotherm, (**b**) Am/TEMPO-CNF pore size distribution, (**c**) FTIR spectra of TEMPO-CNF, Am-CNF, and Am/TEMPO-CNF, and (**d**) TGA-DTG thermograph of TEMPO-CNF, Am-CNF, and Am/TEMPO-CNF.

One of the most essential factors in an adsorption material’s effectiveness is its specific surface area. There is a positive correlation between the specific surface area and porosity of a material and its adsorption capacity. The adsorbent’s surface is adorned with active chemical functional groups, including the quaternary amine group, electrostatic bonding sites, and hydroxyl groups. Hence, the adsorption capacity exhibits a positive correlation with the augmentation of the specific surface area. These groups can establish stable interactions with pollutants, therefore, augmenting the adsorption capacity. According to the IUPAC classification of adsorption isotherms [23], Am/TEMPO-CNF’s adsorption isotherms are of type-IV with the presence of a type H3 hysteresis loop (Figure 1a). This hysteresis loop suggests the coexistence of mesopores and macropores with plate-like layered structure inside the aerogel structure (Figure 1b) [24]. The total pore volume is 0.05305 cc/g for pores diameter smaller than 79.8 nm and the average pores diameter is 3.314 nm.

The FTIR spectra of Am/TEMPO-CNF, Am-CNF, and TEMPO-CNF is presented in Figure 1c. A new peak appeared at 1480 cm^−1^ in both Am-CNF and Am/TEMPO-CNF which might represent the triethyl group of quaternary ammonium [25]. The peak at 1640 cm^−1^ might be due to the vibration of a quaternary nitrogen bond or bound water. Also, the observed splitting of the peak at 2890 and 2950 cm^−1^ in both Am-CNF and Am/TEMPO-CNF samples might be attributed to the presence of a quaternary ammonium group [26,27]. This splitting is likely caused by the out-of-plane bending of the C–H stretching in the ethyl group. A noticeable peak at 1610 cm^−1^ in the TEMPO-CNF spectra is attributed to carbonyl groups, distinguishing it from other substances [28].

The thermogravimetric analysis has been performed to study the influence of temperature on Am/TEMPO-CNF, TEMPO-CNF, and Am-CNF (Figure 1d). All CNFs exhibited 10% sharp weight loss at 100 °C, which could be due to moisture loss. Additionally, it is possible that the low molecular weight of the lignocellulose nanofiber, caused by the chemical treatment with TEMPO, contributed to the breakdown process at lower temperatures [29]. From 100 to 200 °C, all three CNFs are stable without a significant weight loss after which a maximum weight loss (Tmax) is observed at 350 °C, 340 °C, and 310 °C for Am/TEMPO-CNF, Am-CNF, and TEMPO-CNF, respectively, which is potentially associated with the breakage of cellulose glycosidic linkages [30]. A slight reduction in weight was noticed when the temperature increased from 360 °C to 800 °C. This weight loss can be attributed to the carbonation process, wherein the glucose present in the aerogels undergoes additional pyrolysis, resulting in the formation of low-molecular-weight compounds [31]. A significant decrease in the thermal degradation point with higher residual amount of TEMPO-CNF comparing to Am/TEMPO-CNF and Am-CNF aerogels occurred due to the formation of sodium carboxylate groups from the C6 primary hydroxyls of cellulose nanofibril surfaces by TEMPO-mediated oxidation [32].

**Figure 2:**
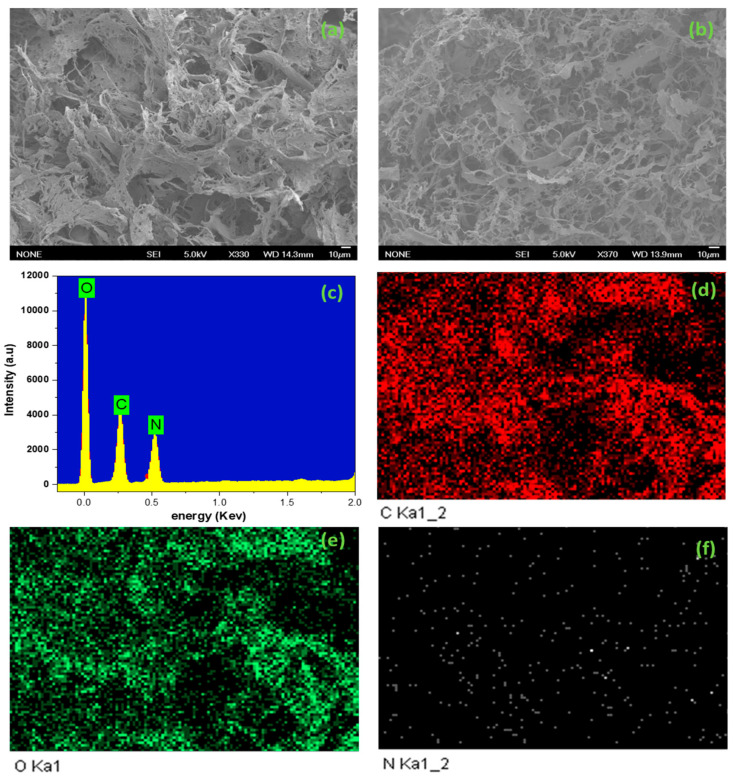
Fe-SEM micrograph of (**a**) Am-CNF, (**b**) Am/TEMPO-CNF, and (**c**–**f**) Electron image and EDS of Am/TEMPO-CNF.

Scanning electron microscopy has been used to study the morphology of Am-CNF and Am/TEMPO-CNF. A clear difference can be observed between morphology of Am-CNF and Am/TEMPO-CNF (Figure 2a,b). The surface of Am-CNF is smooth and denser compared to Am/TEMPO-CNF. The Am/TEMPO-CNF structure appears to be more porous compared to Am-CNF, which might be due to TEMPO oxidation of nanocellulose. The BET surface area of Am/TEMPO-CNF is 20.75 m^2^/g, which is comparatively higher than the BET surface area of Am-CNF (9.18 m^2^/g) as shown in Table 1. The mesoporous structure of Am/TEMPO-CNF favors antibiotics removal by offering more active sites for their adsorption [24]. The primary process that enables fibrillation in TEMPO-CNF involves the introduction of a negative surface charge to the fibrils. The phenomenon of electrostatic repulsion forces plays a crucial role in the liberation of individual fibrils and the maintenance of their stability within a colloidal dispersion. When the carboxyl group concentration reaches a sufficient level, a gentle mechanical treatment is capable of inducing fibrillation in the pretreated cellulose fibers, though to a limited extent [33]. The efficacy of the modification was also validated using EDX spectrum analysis, which demonstrates the effective dispersion of quaternary amine groups on the Am/TEMPO-CNF surface (Figure 2c–f).

### 2.2. Adsorption Studies

#### 2.2.1. pH Influence

**Figure 3:**
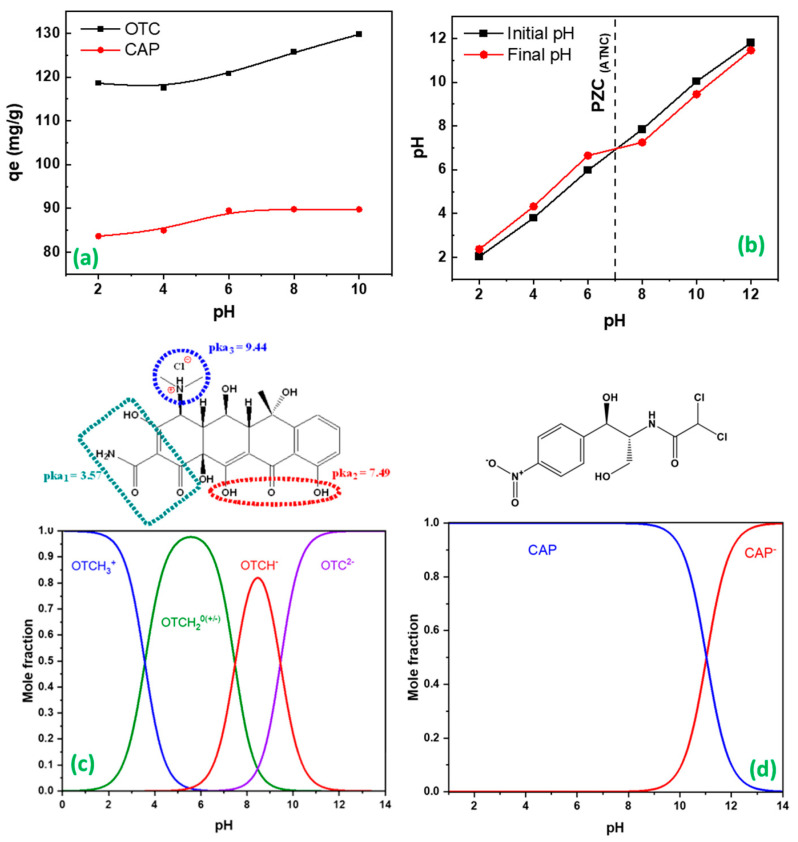
(**a**) Effect of pH on the adsorption capacity, (**b**) point of zero charge (PZC), (**c**) OTC structure with pH-dependent speciation curve, and (**d**) CAP structure with pH-dependent speciation curve.

The overall adsorption mechanism is primarily dependent on the pH, as it influences the functional groups on the adsorbent surface and antibiotic speciation. The trend of OTC and CAP adsorption at different pH is depicted in Figure 3a. The adsorption of both OTC and CAP increased significantly starting from pH 4. The PZC of Am/TEMPO-CNF was determined to be 7.0 (Figure 3b). The surface of Am/TEMPO-CNF has a persistent quaternary ammonium cation at all pH ranges, which makes it highly active in the presence of any anionic species at a pH higher than 7.0. As mentioned in the OTC speciation curve (Figure 3c), OTC and Am/TEMPO-CNF both have a positive charge (OTCH^3+^) when the pH is less than 7.0 due to the presence of the ammonium cations causing electrostatic repulsion and decreasing the adsorption capacity. When the solution pH passes 7.0, OTC’s surface charge turns to negative (OTCH^−^ and OTC^2−^) due to the presence of the phenoxide ion, which will bind strongly to the quaternary ammonium cation of Am/TEMPO-CNF through an electrostatic interaction in addition to hydrogen bonding. Based on that mechanism, an improved adsorption trend was observed at pH higher than 6 [34,35]. The improved adsorption trend at higher pH could be attributed to the electrostatic interaction and hydrogen bonding and decreased electrostatic repulsion.

From the CAP speciation curve (Figure 3d), the net surface charge of CAP is almost neutral while Am/TEMPO-CNF is positive when the solution pH is less than 8.0, leading to a reduction in electrostatic interaction and adsorption capacity. The maximum adsorption of CAP is exhibited between pH 7 and 8, which might be due to the fact that after pH 7 the Am/TEMPO-CNF surface charge becomes positively charged and the CAP surface charge began to change to negative (CAP^—^) from pH 8. This might be causing an increase in the electrostatic attractions, hydrogen bonding, and, accordingly, the adsorption strength. After pH 8, an alkaline hydrolytic cleavage of the CAP amide group occurs producing 2-Amino-1-(4-nitrophenyl)-1,3-propanediol and 2,2-dichloroacetate fragments [36,37].

#### 2.2.2. Adsorption Kinetics

**Figure 4:**
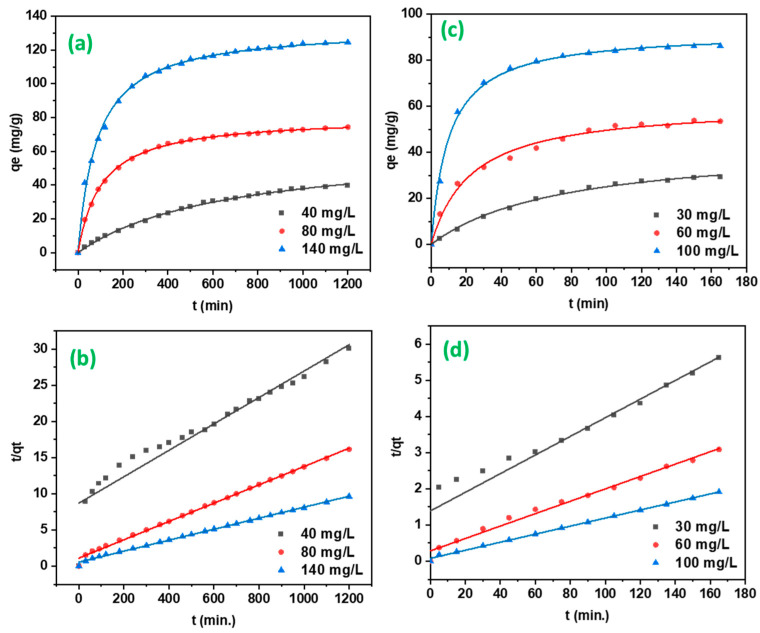
The adsorption kinetics curves of OTC and CAP using Am/TEMPO-CNF at room temperature: (**a**) OTC pseudo-second order non-linear fit, (**b**) OTC pseudo-second order linear fit, (**c**) CAP pseudo-second order non-linear fit, and (**d**) CAP pseudo-second order linear fit.

Adsorption kinetics provide insightful knowledge regarding the mechanism of sorption and the rate of reaction occurring on the adsorbent’s surface during adsorption. The adsorption data for OTC and CAP was fitted into pseudo-first order (PSO-1) and pseudo-second order (PSO-2) models to depict the behavior of both antibiotics on different contact times as represented in Figure 4 and Table 2. The outcomes were best fitted to PSO-2 as the R^2^ values of OTC (0.33 to 0.50) and CAP (0.06 to 0.33) for PSO-1 were lower compared to the R^2^ value of OTC (0.995 to 0.997) and CAP for PSO-2 (0.987 to 0.998). This finding may suggest that chemisorption is the dominant adsorption mechanism in the adsorption of OTC and CAP via Am/TEMPO-CNF [38]. The qe of OTC (39.76 to 131.5 mg/g) was higher compared to the qe of CAP (27.012 to 89.928 mg/g) using Am/TEMPO-CNF. CAP adsorption attained equilibrium after just one hour. However, the OTC attained its adsorption equilibrium at 10 h after a substantial increase in the adsorption of OTC using Am/TEMPO-CNF. The adsorption of OTC and CAP was fast during the initial time period, after which it slowed down and reached its equilibrium. This finding is consistent with antibiotics rapidly diffusing into the adsorbent’s micropores during the initial stage of the process. The adsorption sites are then occupied in a step-by-step fashion until an equilibrium is reached [39].

#### 2.2.3. Adsorption Isotherms

**Figure 5:**
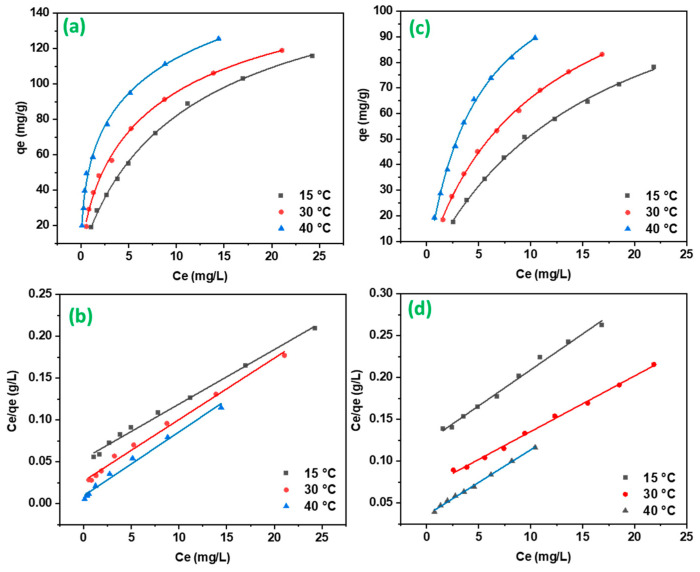
The adsorption isotherm curves of OTC and CAP using Am/TEMPO-CNF at different temperatures: (**a**) OTC Langmuir non-linear fit, (**b**) OTC Langmuir linear fit, (**c**) CAP Langmuir non-linear fit, and (**d**) CAP Langmuir linear fit.

Adsorption isotherms for the adsorbate ions at different starting concentrations allowed us to calculate maximal adsorption capacity and determine the adsorbate–adsorbent relationship. The Langmuir and Freundlich models are often used in sorption isotherm investigations. Antibiotic molecules constantly diffuse from the area with greater concentration (the solution) to the area with lower concentration (the adsorbent) until the system reaches equilibrium, which is consistent with a change in the concentration gradient [13]. The influence of different initial concentrations (C_o_) of OTC (20 to 140 mg/L) and CAP (20 to 100 mg/L) at three different temperatures (15, 30, and 40 °C) is observed Figure 5. The adsorption capacity of both OTC and CAP was increased with an increase in the initial concentration and temperature. The maximum adsorption capacity q_max_ of OTC was ranged from 130.71 to 153.13 mg/g and CAP spanned from 116.14 to 150.15 mg/g, with maximum adsorption at 40 °C (Table 3). The adsorption concentrations of both antibiotics fitted well to Langmuir, confirming the monolayer adsorption was primary alongside minimal occurrence of multilayer chemical adsorption [40].

The adsorption potential of Am/TEMPO-CNF for OTC and CAP is compared with different adsorbents in Table 4. The highest adsorption capacity among these comparators is 150.15 and 153.13, exhibited by Am/TEMPO-CNF for CAP and OTC removal, respectively. Furthermore, the fabrication technique of Am/TEMPO-CNF is a simple and economical substitute for other adsorbents.

#### 2.2.4. Removal Mechanism

**Figure 6:**
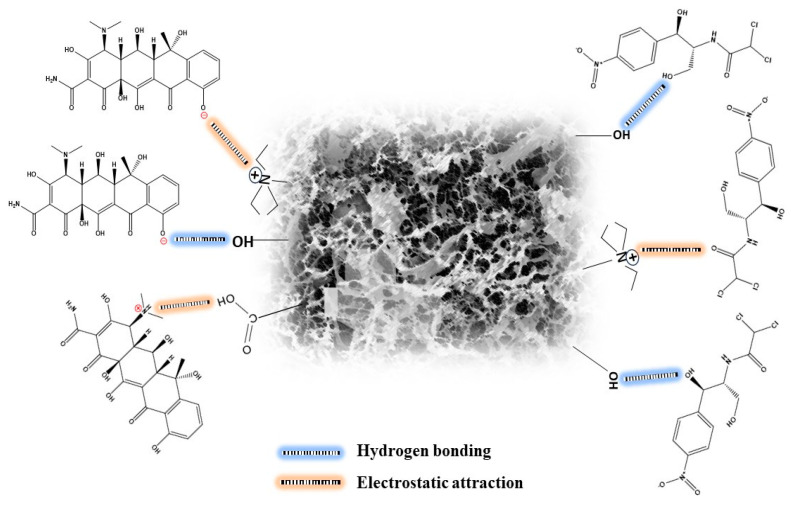
Removal mechanism of OTC and CAP using Am/TEMPO-CNF.

The results of our study indicate that the adsorption process of both antibiotics by Am/TEMPO-CNF involves mainly hydrogen bonding and electrostatic attraction (Figure 6). Hydrogen bonding could occur between the carboxyl and hydroxyl group of Am/TEMPO-CNF and the hydroxyl group and amide group of CAP [48]. The observed high adsorption capacity of CAP and OTC may be attributed to the occurrence of electrostatic interactions with the anionic sites of CAP and the phenolic diketone moiety anion of OTC, which possess a negative charge. The hydrogen bonding between -OH and -COOH groups in the adsorbent and -OH amide groups and tertiary amine (-N-(CH_3_)_2_) groups in the OTC is involved in the removal of OTC [5].

#### 2.2.5. Adsorption Thermodynamics

The adsorption potential of Am/TEMPO-CNF for OTC and CAP was increased with the temperature (Table 5). The value of ΔH° was positive (29.981 and 32.060 KJ·mol^−1^) for both OTC and CAP, which indicates their adsorption is an endothermic process. The negative values of ΔG° (−2.25753, −4.15437, and −5.60633 KJ·mol^−1^) and (−3.20431, −4.88925, and −6.87970 KJ·mol^−1^) for OTC and CAP, respectively, reveals the adsorption process was via spontaneous reaction. The observed correlation between increasing temperature and a more negative ΔG value suggests that higher temperatures are conducive to the removal of antibiotics. Both the OTC and CAP antibiotics had positive ΔS° values of 112.1152 and 122.2296 J·mol^−1^·K^−1^, respectively, suggesting a significant level of interaction between these antibiotics and Am/TEMPO-CNF [49].

#### 2.2.6. Regeneration Study

**Figure 7:**
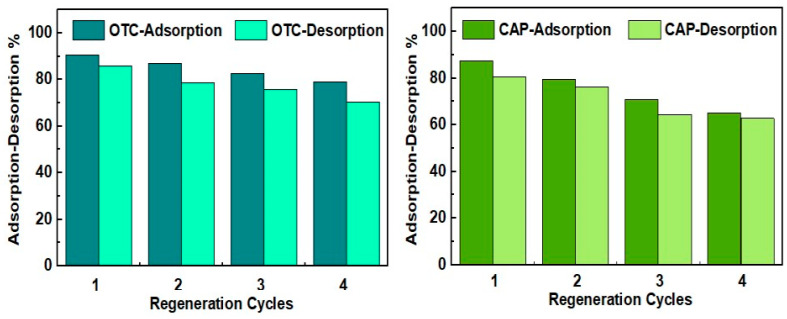
Regeneration cycles of Am/TEMPO-CNF for OTC and CAP.

Good reusability is equally crucial in addition to adsorbing capacity and a fast adsorption rate for an adsorbent to be considered optimal. The reusability of Am/TEMPO-CNF adsorption was assessed over four consecutive adsorption–desorption cycles using a adsorbent solution of 0.01 mol L^−1^ NaOH. A decreased trend in the adsorption–desorption % of OTC and CAP has been observed with each cycle, which might be due to the saturation of functional groups on the Am/TEMPO-CNF’s surface and minimal structural damage [50]. The adsorption potential of Am/TEMPO-CNF for OTC and CAP decreased from 90.64% and 85.87% to 79.04% and 70.36%, respectively. The Am/TEMPO-CNF could remove more than 70% of both OTC and CAP after four regeneration cycles. The findings suggest that the Am/TEMPO-CNF may be effectively reused for up to four regeneration cycles (Figure 7). This demonstrates that the Am/TEMPO-CNF has outstanding regeneration capabilities, making it a promising and cost-effective adsorbent for the removal of antibiotics.

## 3. Conclusions

This research study highlights the effectiveness of Am/TEMPO-CNF as a promising adsorbent for the removal of oxytetracycline (OTC) and chloramphenicol (CAP) from water-based solutions. The solution pH was kept constant throughout the studies, with CAP and OTC at around 7 and 8 pH, respectively, which is an important factor in real-world wastewater treatment procedures. The analytical techniques including elemental analysis, FTIR, BET, TGA, and SEM confirm the successful fabrication of modified Am/TEMPO-CNF. The noteworthy adsorption capabilities of Am/TEMPO-CNF for these antibiotics are highlighted by the maximal adsorption capacities (Qmax) of 153.13 mg/g for OTC and 150.15 mg/g for CAP. Higher temperatures are favorable for the elimination of both antibiotics, as shown by the observed correlation between increased temperature and a greater negative ΔG value. The adsorption kinetics of both antibiotics fitted well to PSO-2, indicating the adsorption is mainly through chemical interactions including electrostatic attraction and hydrogen bonding due to the presence of -OH, -COOH, and quaternary amine groups on the adsorbent and -OH, -CONH_2_, and -N-(CH_3_)_2_ groups on the adsorbate. The recyclability investigation further confirms that Am/TEMPO-CNF can be practically used for several cycles with minimal degradation of adsorption capability. Further studies can be conducted using life cycle assessment (LCA) to determine the environmental impact and cost–benefit of utilizing Am/TEMPO-CNF to remediate various antibiotics and pollutants, which is very important in assessing the viability of field-scale applications and the possibility for commercial consideration in the future.

## 4. Materials and Methods

### 4.1. Materials

The chemicals used in this study are of analytical grade. Oxytetracycline (OTC, Thermo scientific, CAS: 79-57-2, empirical formula C_22_H_26_N_2_Na_2_O_11_, MW: 460.434 g/mol) was obtained from Thermo Scientific, USA. Chloramphenicol (CAP, bioWORLD, CAS: 56-75-7, empirical formula C_11_H_12_Cl_2_N_2_O_5_, MW: 323.132 g/mol) was purchased from bioWORLD. The TEMPO-CNF and unmodified CNF (3% aqueous slurry) were both obtained from The University of Maine. Hydrochloric acid (HCl), 37.1% certified ACS plus, and Triethylamine (TEA), 99% reagent grade, was purchased from Fisher Chemical. Sodium hydroxide bioxtra, ≥98% pellets (anhydrous). Epichlorohydrin (EPH), Aldrich, 99% acroseal was purchased from Acros Organics. Highly pure deionized water of 17.8 megaohm was used for the preparation of synthetic wastewater.

### 4.2. Aminated Cellulose Nanofiber (Am-CNF) Synthesis

Am-CNF was synthesized from regenerated CNF according to the previously reported work [51]. Briefly, 10 g of NaOH pellets were added to 100 mL of CNF (3% aqueous slurry) in a 250 mL volumetric flask under mechanical stirring. The alkaline CNF was placed in a cooled bath (−5 °C) until the formation of transparent CNF. Then, DI water was added to the transparent CNF gel and was neutralized using HCl to pH 7. After neutralization, regenerated CNF was obtained after blending CNF at high speed of 25,000 rpm using a mechanical blender (Vitamix 5200) for 20 min. The regenerated CNF was washed with DI water and vacuum filtered using a filter paper (Whatman grade 42). An amount of 25% of the previously prepared regenerated CNF was added to 100 mL of DI water. Then, 14 mL of TEA and 7.8 mL of EPH were added in this solution dropwise under stirring. The solution was refluxed for 24 h at 80 °C under magnetic stirring. The next day, the solution was neutralized, filtered, and washed with DI water until pH 7 was obtained. The Am-CNF hydrogel was then freeze dried to obtain Am-CNF aerogel.

### 4.3. Aminated/TEMPO Cellulose Nanofiber (Am/TEMPO-CNF) Aerogel Synthesis

**Scheme 1:**
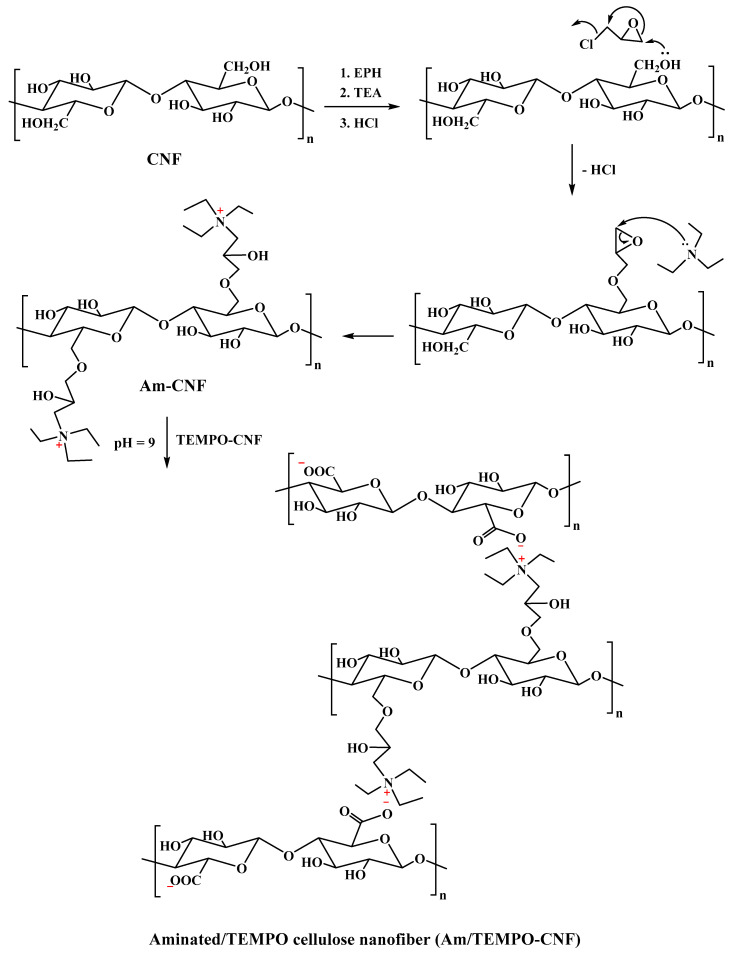
Synthesis mechanism of Am/TEMPO-CNF aerogel.

The step-by-step synthesis mechanism of *Am/TEMPO-CNF* aerogel is depicted in Scheme 1. Briefly, 1 g of the prepared Am-CNF was dispersed in 100 mL of DI water in a 250 mL volumetric flask and 2 g of TEMPO-CNF was added dropwise in the solution with continuous stirring. The pH of the solution was adjusted to 9 using 0.1 M NaOH. The mixture was then neutralized, vacuum filtered, and washed using DI water until the pH 7 was attained. The obtained hydrogel composite was placed in a freeze dryer for 4 days to obtain Am/TEMPO-CNF aerogel.

### 4.4. Aerogel Characterization

The characterization of Am/TEMPO-CNF was conducted to study its physiochemical characteristics. Carbon, hydrogen, nitrogen content, and oxygen (by subtraction) were measured with a CE-440 Elemental Analyzer (Exeter Analytical, North Chelmsford, MA, USA). The surface morphology was investigated using FE-SEM (JEOL JSM-6500F). The thermal analysis (TGA-DTG) was performed using the thermogravimetric analyzer (SDT Q600 series). The Quantachrome Autosorb iQ gar sorption analyzer (Quantachrome ASIC0500-5, West Palm Beach, FL, USA) was used to measure pore volume and surface area of Am/TEMPO-CNF.

### 4.5. Point of Zero Charge (PZC)

The PZC of Am/TEMPO-CNF was measured using pH drift method. NaCl solutions, 20 mL of 0.01 M, were prepared with a pH 2, 4, 6, 8, 10, 12 using 0.1 M HCl and NaOH solutions. Before adding Am/TEMPO-CNF, the NaCl solutions were purged with N_2_ gas to remove the dissolved CO_2_. An amount of 20 mg of Am/TEMPO-CNF was immersed in each NaCl solution and agitated for 24 h at 250 rpm. The Am/TEMPO-CNF was filtered, and the pH of all solutions was measured again. The initial and final pH values were plotted in the graph to obtain the PZC of Am/TEMPO-CNF.

### 4.6. Adsorption Experiments

#### 4.6.1. Effect of pH

The pH influence on the adsorption of OTC and CAP was studied by adjusting the pH range of OTC and CAP solutions from 2 to 12 using NaOH or HCl at 25 °C. An amount of 10 mg of Am/TEMPO-CNF was added to 10 mL of 100 ppm CAP and 140 ppm of OTC solutions having different pH ranges at contact times of 1 h and 10 h, respectively. Both samples were filtered using 0.22 µm syringe after their respective contact times.

#### 4.6.2. Adsorption Kinetics

The influence of time on the removal of OTC and CAP by Am/TEMPO-CNF was examined. An amount of 10 mg of Am/TEMPO-CNF was added into 10 mL of OTC (40, 80, and 140 ppm) and CAP (30, 60, and 100 ppm) solutions at 25 °C and agitated at 250 rpm for 10 to 1200 min. and 10 to 165 min., respectively. The pH of the solutions was maintained at 8 for OTC and 7 for CAP. The solutions were filtered using 0.22 µm (pore size) filter paper. The OTC and CAP concentration before and after the adsorption experiment were measured using a spectrophotometer (Azzota SM1800PC UV-Vis) at wavelengths of 360 nm and 278 nm, respectively. The removal efficiency of Am/TEMPO-CNF for OTC and CAP was calculated using following equation.

(1) $$\%\;\mathrm{removal}=\left(\frac{C_{o}-C_{e}}{C_{o}}\right)\times 100$$

The sorption capacity of Am/TEMPO-CNF for both antibiotics was determined using the following equation.

(2) $$\mathrm{qt}=\frac{\left(C_{o}-C_{t}\right)V}{m}$$

The antibiotic concentration at time t is denoted by C_t_ (mg/L), initial concentration is C_o_ (mg/L), adsorbent mass is denoted by m (g), and V is volume (L).

#### 4.6.3. Adsorption Isotherms

The effect of temperature on adsorption was evaluated at 15, 30, and 40 °C. The isothermal solutions were prepared by adding 10 mg Am/TEMPO-CNF to 10 mL of OTC (20 to 140 ppm) and CAP (20 to 100 ppm) at pH 8 and 7, respectively. After filtering, samples were analyzed for adsorption via UV/Vis spectrophotometry. The adsorption data were fitted to linear and non-linear Freundlich and Langmuir isotherms. The equations for both adsorption kinetics and adsorption isotherms are presented in Table 6.

#### 4.6.4. Adsorption Thermodynamics

Examining the temperature dependence of adsorption at different temperatures allowed us to examine the impact of temperature on the change in internal energy. For this purpose, the OTC and CAP adsorption at different temperatures 15, 30, and 45 °C is examined. Adsorption-related thermodynamic parameter fluctuations were determined using the Van’t Hoff equations:

(3) $$\Delta G=-{\mathrm{RTlnK}}_{d}$$

(4) $${\mathrm{lnK}}_{d}=\frac{\Delta S}{R}-\frac{\Delta H}{\mathrm{RT}}$$

(5) $$K_{d}=\frac{q_{e}}{\begin{array}{c} C_{e} \end{array}}$$

R is gas constant (8.314 J K^−1^), ΔH, ΔG, ΔS, and K_d_ are enthalpy (KJ·mol^−1^), heat capacity (kJ·mol^−1^), entropy (J·mol^−1^ K^−1^), and distribution coefficient, respectively.

#### 4.6.5. Reusability Study

Recyclability was studied with 10 mg of Am/TEMPO-CNF added to 10 mL of 140 ppm OTC and 100 ppm CAP solution for 10 h and 1 h, respectively. After adsorption, each Am/TEMPO-CNF sample was filtered and immersed in 10 mL of 0.1 M NaOH solution and agitated at 250 rpm for 2 h for the desorption of antibiotics. After desorption, Am/TEMPO-CNF was washed, filtered, and dried for the next cycles. The adsorption–desorption experiment was repeated 4 times. The desorption % of both antibiotics was calculated using following equation:

(6) $$\%\;\mathrm{Desorption}=\left(\frac{C_{o}\mathrm{des}}{C_{\mathrm{sorb}}}\right)\times 100$$

C_sorb_ is adsorbed antibiotic concentration and C_o_ is antibiotic initial concentration.

## Figures and Tables

**Table 1:** Elemental analysis and surface area of Am/TEMPO-CNF, Am-CNF, and CNF.

Sample	Elemental Analysis				Surface Area (m^2^/g)
	%C	%H	%N	%R	
Am/TEMPO-CNF	41.54	6.38	1.26	50.82	20.75
Am-CNF	40.06	7.93	2.93	49.09	9.18
CNF	41.89	6.02	0.00	52.09	2.82

**Table 2:** The adsorption kinetic values for OTC (40, 80, and 140 mg/L) and CAP (30, 60, and 100 mg/L) at initial concentration, 0.01 (g/L) Am/TEMPO-CNF dosage, and temperature 30 °C.

Models	C_o_ (mg/L)	OTC			CAP		
		40	80	140	30	60	100
Pseudo-first order	K_1_ (1/min)	0.00304	0.0046	0.00422	0.01291	0.02182	0.02167
	q_e_ (mg/g)	21.598	29.216	41.412	11.578	18.36	16.030
	R^2^	0.47208	0.50764	0.33198	0.06063	0.33219	0.22756
Pseudo-second order	K_2_ (g/mg/min)	0.00025	0.00014	0.00010	0.00537	0.00102	0.00154
	q_e_ (mg/g)	39.761	79.051	131.578	27.012	58.411	89.928
	R^2^	0.995	0.996	0.997	0.998	0.987	0.997

**Table 3:** Langmuir and Freundlich models parameters for OTC and CAP adsorption at three selected temperatures (15, 30, and 40 °C).

Models	Temp (°C)	OTC			CAP		
		15	30	40	15	30	40
Langmuir	K_1_ (L/mg)	0.00002	0.2727	0.1218	0.0700	0.00002	0.0969
	q_max_ (mg/g)	130.71	136.05	153.13	116.14	128.20	150.15
	R^2^	0.986	0.992	0.993	0.991	0.997	0.996
Freundlich	KF ([mg g^−1^(Lmg^−1^)1/n])	20.675	31.668	51.90	10.729	14.884	24.635
	n	1.735	2.11	2.74	1.460	1.630	1.686
	R^2^	0.984	0.967	0.974	0.984	0.981	0.981

**Table 4:** Comparison of Am/TEMPO-CNF adsorption capacity for OTC and CAP with other adsorbents.

Adsorbent	Adsorbate	pH	q_max_ (mg/g)	Reference
Am/TEMPO-CNF	CAP	7.0	150.15	This study
corn stalk biomass fiber		6.5	50.17	[41]
sodium bicarbonate (NaHCO_3_)-impregnated coconut husk-activated carbon (CHAC)		-	4.0	[42]
AC from eucalyptus wood		4–4.5	21	[43]
Novel magnetic molecularly imprinted polymers (MMIPs)		-	52.6	[44]
Am/TEMPO-CNF	OTC	8	153.1	This study
kaolinite		5.5	15.2	[45]
magnetic layered double hydroxide (MLDH)		7	127.7	[46]
Graphene oxide functionalized magnetic particles		-	45.0	[47]
Posidonia oceanica		6	11.8	[34]

**Table 5:** Thermodynamics factors estimated for the OTC and CAP adsorption at varied temperatures.

Adsorbate	T (°C)	Q_e_ (mg/g)	$K_{d}=\frac{q_{e}}{C_{e}}$	$lnK_{d}$	ΔG° (KJ·mol^−1^)	ΔH° (KJ·mol^−1^)	ΔS° (J·mol^−1^·K^−1^)
OTC	15	39.26	2.56597	0.94233	−2.25753	29.98155	112.1152
	30	22.9	5.19816	1.64830	−4.15437		
	40	15.0	8.32711	2.11951	−5.60633		
CAP	15	20.78	3.81231	1.33823	−3.20431	32.06048	122.2296
	30	12.43	6.96460	1.94084	−4.88925		
	40	6.9	13.4927	2.60215	−6.87970		

**Table 6:** Kinetics and Adsorption Isotherms Models.

Model	Equation
Langmuir linear	$\frac{\mathrm{Ce}}{\mathrm{qe}}=\frac{1}{\mathrm{Kl}\;\mathrm{qmax}}+\frac{\mathrm{Ce}}{\mathrm{qmax}}$
Langmuir non-linear	$\mathrm{Qe}=\frac{\mathrm{QmKlCe}}{1+\mathrm{KlCe}}$
Freundlich linear	$\ln\left(\mathrm{qe}\right)=\left[\ln\left(\mathrm{Kf}\right)+\left(\frac{1}{n}\right)\ln\left(\mathrm{Ce}\right)\right]$
Freundlich non-linear	$\mathrm{Qe}={\mathrm{KfCe}}^{1/n}$
Pseudo-first order non-linear	$\mathrm{Qt}=\mathrm{Qe}\left(1-e^{-k1t}\right)$
Pseudo-first order linear	$\log\left(\mathrm{qe}-\mathrm{qt}\right)=\left[\log\left(\mathrm{qe}\right)-\left(\frac{k_{1}}{2.303}\right)\times t\right]$
Pseudo-second order non-linear	$\mathrm{Qt}=\frac{k_{2}{\mathrm{tQe}}^{2}}{1+k_{2}\mathrm{tQe}}$
Pseudo-second order linear	$\frac{t}{\mathrm{qt}}=\frac{1}{k_{2}{\left(\mathrm{qe}\right)}^{2}}+\frac{t}{\mathrm{qe}}$

Where qe (mg/g) is the equilibrium adsorption capacity, qt (mg/g) is the adsorption capacity at time t, k_1_ (min^−1^) is the rate constant of pseudo-first order adsorption, and k_2_ (g/(mg min)) is the rate constant of pseudo-second order adsorption. Ce (mg/L) is the antibiotic concentration at equilibrium, qmax (mg/g) is the maximum adsorption capacity, Kl is the Langmuir adsorption constant related to adsorption energy, and Kf and n are the Freundlich adsorption constants which indicate the capacity and intensity of the adsorption, respectively.

## Data Availability

Data are contained within the article.

## References

[1] Chu Y., Li S., Xie P., Chen X., Li X., Ho S.-H. (2023). New insight into the concentration-dependent removal of multiple antibiotics by Chlorella sorokiniana. Bioresour. Technol..

[2] Chen J., Sun R., Pan C., Sun Y., Mai B., Li Q.X. (2020). Antibiotics and food safety in aquaculture. J. Agric. Food Chem..

[3] Chen G., He S., Shi G., Ma Y., Ruan C., Jin X., Chen Q., Liu X., Dai H., Chen X. (2021). In-situ immobilization of ZIF-67 on wood aerogel for effective removal of tetracycline from water. Chem. Eng. J..

[4] Ruan C., Chen G., Ma Y., Du C., He C., Liu X., Jin X., Chen Q., He S., Huang Y. (2023). PVA-assisted CNCs/SiO_2_ composite aerogel for efficient sorption of ciprofloxacin. J. Colloid Interface Sci..

[5] Cha B., Kim N., Yea Y., Han J., Yoon Y., Kim S., Park C.M. (2023). Comprehensive evaluation of antibiotic tetracycline and oxytetracycline removal by Fe-metal organic framework/biopolymer-clay hydrogel. Ceram. Int..

[6] Zhou H., Jiao G., Li X., Gao C., Zhang Y., Hashan D., Liu J., She D. (2023). High capacity adsorption of oxytetracycline by lignin-based carbon with mesoporous structure: Adsorption behavior and mechanism. Int. J. Biol. Macromol..

[7] Singh R., Singh A.P., Kumar S., Giri B.S., Kim K.-H. (2019). Antibiotic resistance in major rivers in the world: A systematic review on occurrence, emergence, and management strategies. J. Clean. Prod..

[8] Ben W., Pan X., Qiang Z. (2013). Occurrence and partition of antibiotics in the liquid and solid phases of swine wastewater from concentrated animal feeding operations in Shandong Province, China. Environ. Sci. Process. Impacts.

[9] Liu X., Pei Y., Cao M., Yang H., Li Y. (2023). Magnetic CuFe_2_O_4_ nanoparticles anchored on N-doped carbon for activated peroxymonosulfate removal of oxytetracycline from water: Radical and non-radical pathways. Chemosphere.

[10] Jin W., Feng J., Xing W., Tang W. (2023). Significantly Enhanced Electrocatalytic Reduction of Chloramphenicol from Water Mediated by Fe–F–N Co-doped Carbon Materials. ACS EST Water.

[11] Lv Y., Han L., Shen J., Li J., Dong H., Hu J., He Y., Li Y. (2024). Impact of environmental factors on the removal of chloramphenicol by zero-valent iron and pyrite mixture. Sep. Purif. Technol..

[12] Mokni S., Tlili M., Jedidi N., Hassen A. (2022). Applicability of electrocoagulation process to the treatment of Ofloxacin and Chloramphenicol in aqueous media: Removal mechanism and antibacterial activity. J. Water Process Eng..

[13] Nguyen L.M., Nguyen N.T.T., Nguyen T.T.T., Nguyen T.T., Nguyen D.T.C., Tran T.V. (2022). Occurrence, toxicity and adsorptive removal of the chloramphenicol antibiotic in water: A review. Environ. Chem. Lett..

[14] Zhao J., Yuan X., Wu X., Liu L., Guo H., Xu K., Zhang L., Du G. (2023). Preparation of nanocellulose-based aerogel and its research progress in wastewater treatment. Molecules.

[15] Yao Q., Fan B., Xiong Y., Jin C., Sun Q., Sheng C. (2017). 3D assembly based on 2D structure of cellulose nanofibril/graphene oxide hybrid aerogel for adsorptive removal of antibiotics in water. Sci. Rep..

[16] Xia C., Huang H., Liang D., Xie Y., Kong F., Yang Q., Fu J., Dou Z., Zhang Q., Meng Z. (2022). Adsorption of tetracycline hydrochloride on layered double hydroxide loaded carbon nanotubes and site energy distribution analysis. Chem. Eng. J..

[17] Zhao X., Gao X., Ding R., Huang H., Gao X., Liu B. (2023). Post-synthesis introduction of dual functional groups in metal–organic framework for enhanced adsorption of moxifloxacin antibiotic. J. Colloid Interface Sci..

[18] Pang W., Wang Y., Li S., Luo Y., Wang G., Hou J., Han T., Gao Z., Guo Q., Zhou H. (2023). Novel magnetic graphoxide/biochar composite derived from tea for multiple SAs and QNs antibiotics removal in water. Environ. Sci. Pollut. Res..

[19] Selvaraj R., Prabhu D., Kumar P.S., Rangasamy G., Murugesan G., Rajesh M., Goveas L.C., Varadavenkatesan T., Samanth A., Balakrishnaraja R. (2023). Adsorptive removal of tetracycline from aqueous solutions using magnetic Fe_2_O_3_/activated carbon prepared from Cynometra ramiflora fruit waste. Chemosphere.

[20] Ahmad A., Kamaruddin M.A., HPS A.K., Yahya E.B., Muhammad S., Rizal S., Ahmad M.I., Surya I., Abdullah C. (2023). Recent Advances in Nanocellulose Aerogels for Efficient Heavy Metal and Dye Removal. Gels.

[21] Nan Y., Gomez-Maldonado D., Iglesias M.C., Whitehead D.C., Peresin M.S. (2023). Valorized soybean hulls as TEMPO-oxidized cellulose nanofibril and polyethylenimine composite hydrogels and their potential removal of water pollutants. Cellulose.

[22] Do N.H., Truong B.Y., Nguyen P.T., Le K.A., Duong H.M., Le P.K. (2022). Composite aerogels of TEMPO-oxidized pineapple leaf pulp and chitosan for dyes removal. Sep. Purif. Technol..

[23] Alothman Z. (2012). A Review: Fundamental Aspects of Silicate Mesoporous Materials. Materials.

[24] Van Hoomissen D.J., Vyas S. (2019). Early events in the reductive dehalogenation of linear perfluoroalkyl substances. Environ. Sci. Technol. Lett..

[25] Pei A., Butchosa N., Berglund L.A., Zhou Q. (2013). Surface quaternized cellulose nanofibrils with high water absorbency and adsorption capacity for anionic dyes. Soft Matter.

[26] Li D., Lee C.-S., Zhang Y., Das R., Akter F., Venkatesan A.K., Hsiao B.S. (2023). Efficient removal of short-chain and long-chain PFAS by cationic nanocellulose. J. Mater. Chem. A.

[27] Saini S., Yücel Falco Ç., Belgacem M.N., Bras J. (2016). Surface cationized cellulose nanofibrils for the production of contact active antimicrobial surfaces. Carbohydr. Polym..

[28] Soni B., Hassan E.B., Mahmoud B. (2015). Chemical isolation and characterization of different cellulose nanofibers from cotton stalks. Carbohydr. Polym..

[29] Mahendra I.P., Wirjosentono B., Tamrin I.H., Mendez J.A. (2019). Thermal and Morphology Properties of Cellulose Nanofiber from TEMPO-oxidized Lower part of Empty Fruit Bunches (LEFB). Open Chem..

[30] Wang X., Zhang Y., Wang S., Jiang H., Liu S., Yao Y., Zhang T., Li Q. (2018). Synthesis and characterization of amine-modified spherical nanocellulose aerogels. J. Mater. Sci..

[31] Zhu W., Chen M., Jang J., Han M., Moon Y., Kim J., You J., Li S., Park T., Kim J. (2024). Amino-functionalized nanocellulose aerogels for the superior adsorption of CO_2_ and separation of CO_2_/CH_4_ mixture gas. Carbohydr. Polym..

[32] Isogai A., Saito T., Fukuzumi H. (2011). TEMPO-oxidized cellulose nanofibers. Nanoscale.

[33] Levanic J., Šenk V.P., Nadrah P., Poljanšek I., Oven P., Haapala A. (2020). Analyzing TEMPO-oxidized cellulose fiber morphology: New insights into optimization of the oxidation process and nanocellulose dispersion quality. ACS Sustain. Chem. Eng..

[34] Ferchichi K., Amdouni N., Chevalier Y., Hbaieb S. (2022). Low-cost Posidonia oceanica bio-adsorbent for efficient removal of antibiotic oxytetracycline from water. Environ. Sci. Pollut. Res..

[35] Punamiya P., Sarkar D., Rakshit S., Datta R. (2013). Effectiveness of Aluminum-based Drinking Water Treatment Residuals as a Novel Sorbent to Remove Tetracyclines from Aqueous Medium. J. Environ. Qual..

[36] Li Y., Zhang J., Liu H. (2018). Removal of Chloramphenicol from Aqueous Solution Using Low-Cost Activated Carbon Prepared from Typha orientalis. Water.

[37] Mitchell S.M., Ullman J.L., Teel A.L., Watts R.J. (2015). Hydrolysis of amphenicol and macrolide antibiotics: Chloramphenicol, florfenicol, spiramycin, and tylosin. Chemosphere.

[38] Peng H., Xiong W., Yang Z., Cao J., Jia M., Xiang Y., Hu Q., Xu Z. (2021). Facile fabrication of three-dimensional hierarchical porous ZIF-L/gelatin aerogel: Highly efficient adsorbent with excellent recyclability towards antibiotics. Chem. Eng. J..

[39] Wu Y., Yue Q., Ren Z., Gao B. (2018). Immobilization of nanoscale zero-valent iron particles (nZVI) with synthesized activated carbon for the adsorption and degradation of Chloramphenicol (CAP). J. Mol. Liq..

[40] Liu H., Wei Y., Luo J., Li T., Wang D., Luo S., Crittenden J.C. (2019). 3D hierarchical porous-structured biochar aerogel for rapid and efficient phenicol antibiotics removal from water. Chem. Eng. J..

[41] Xing W., Liu Q., Wang J., Xia S., Ma L., Lu R., Zhang Y., Huang Y., Wu G. (2021). High selectivity and reusability of biomass-based adsorbent for chloramphenicol removal. Nanomaterials.

[42] Anthonysamy S.I., Yusop M.F.M., Ismail H., Ahmad M.A. (2023). Chloramphenicol Removal from Aqueous Solution Using Sodium Bicarbonate-Impregnated Coconut Husk-Derived Activated Carbon: Optimization and Insight Mechanism Study. Arab. J. Sci. Eng..

[43] Ahmed M.B., Zhou J.L., Ngo H.H., Guo W., Johir M.A.H., Belhaj D. (2017). Competitive sorption affinity of sulfonamides and chloramphenicol antibiotics toward functionalized biochar for water and wastewater treatment. Bioresour. Technol..

[44] Ma W., Dai J., Dai X., Da Z., Yan Y. (2015). Core–shell molecularly imprinted polymers based on magnetic chitosan microspheres for chloramphenicol selective adsorption. Monatshefte Für Chem. Chem. Mon..

[45] Song Y., Sackey E.A., Wang H., Wang H. (2019). Adsorption of oxytetracycline on kaolinite. PLoS ONE.

[46] Smata A., Yoshimura C. (2022). One-step synthesis of magnetic–layered double hydroxide and its application for oxytetracycline removal from water. J. Environ. Chem. Eng..

[47] Lin Y., Xu S., Li J. (2013). Fast and highly efficient tetracyclines removal from environmental waters by graphene oxide functionalized magnetic particles. Chem. Eng. J..

[48] Wei M., Zheng H., Zeng T., Yang J., Fang X., Zhang C. (2021). Porous carbon aerogel derived from bacterial cellulose with prominent potential for efficient removal of antibiotics from the aquatic matrix. Water Sci. Technol..

[49] Chen M., Yan Z., Luan J., Sun X., Liu W., Ke X. (2023). π-π electron-donor-acceptor (EDA) interaction enhancing adsorption of tetracycline on 3D PPY/CMC aerogels. Chem. Eng. J..

[50] Zhang Y., Liu F., Zhong L., Dong Z., Chen C., Xu Z. (2023). Reusable and environmentally friendly cellulose nanofiber/titanium dioxide/chitosan aerogel photocatalyst for efficient degradation of tetracycline. Appl. Surf. Sci..

[51] Elsayed I., Schueneman G.T., El-Giar E.M., Hassan E.B. (2023). Amino-Functionalized Cellulose Nanofiber/Lignosulfonate New Aerogel Adsorbent for the Removal of Dyes and Heavy Metals from Wastewater. Gels.

